# Fitbit Charge HR Wireless Heart Rate Monitor: Validation Study Conducted Under Free-Living Conditions

**DOI:** 10.2196/mhealth.8233

**Published:** 2017-10-20

**Authors:** Alexander Wilhelm Gorny, Seaw Jia Liew, Chuen Seng Tan, Falk Müller-Riemenschneider

**Affiliations:** ^1^ Saw Swee Hock School of Public Health National University of Singapore Singapore Singapore; ^2^ Yong Loo Lin School of Medicine National University of Singapore Singapore Singapore; ^3^ Institute of Social Medicine, Epidemiology and Health Economics Charité University Medical Centre Berlin Germany

**Keywords:** heart rate, photoplethysmography, telemedicine, validation studies

## Abstract

**Background:**

Many modern smart watches and activity trackers feature an optical sensor that estimates the wearer’s heart rate. Recent studies have evaluated the performance of these consumer devices in the laboratory.

**Objective:**

The objective of our study was to examine the accuracy and sensitivity of a common wrist-worn tracker device in measuring heart rates and detecting 1-min bouts of moderate to vigorous physical activity (MVPA) under free-living conditions.

**Methods:**

Ten healthy volunteers were recruited from a large university in Singapore to participate in a limited field test, followed by a month of continuous data collection. During the field test, each participant would wear one Fitbit Charge HR activity tracker and one Polar H6 heart rate monitor. Fitbit measures were accessed at 1-min intervals, while Polar readings were available for 10-s intervals. We derived intraclass correlation coefficients (ICCs) for individual participants comparing heart rate estimates. We applied Centers for Disease Control and Prevention heart rate zone cut-offs to ascertain the sensitivity and specificity of Fitbit in identifying 1-min epochs falling into MVPA heart rate zone.

**Results:**

We collected paired heart rate data for 2509 1-min epochs in 10 individuals under free-living conditions of 3 to 6 hours. The overall ICC comparing 1-min Fitbit measures with average 10-s Polar H6 measures for the same epoch was .83 (95% CI .63-.91). On average, the Fitbit tracker underestimated heart rate measures by −5.96 bpm (standard error, SE=0.18). At the low intensity heart rate zone, the underestimate was smaller at −4.22 bpm (SE=0.15). This underestimate grew to −16.2 bpm (SE=0.74) in the MVPA heart rate zone. Fitbit devices detected 52.9% (192/363) of MVPA heart rate zone epochs correctly. Positive and negative predictive values were 86.1% (192/223) and 92.52% (2115/2286), respectively. During subsequent 1 month of continuous data collection (270 person-days), only 3.9% of 1-min epochs could be categorized as MVPA according to heart rate zones. This measure was affected by decreasing wear time and adherence over the period of follow-up.

**Conclusions:**

Under free-living conditions, Fitbit trackers are affected by significant systematic errors. Improvements in tracker accuracy and sensitivity when measuring MVPA are required before they can be considered for use in the context of exercise prescription to promote better health.

## Introduction

Sedentary behavior, daily step counts, and moderate to vigorous physical activity (MVPA) have been identified as targets for public health intervention [1]. In response to these findings, the practice of physician-directed exercise prescription emerged as a promising strategy to promote the health benefits of physical activity. Under the tagline *Exercise Is Medicine*, clinicians are advocating nonpharmacological interventions in the management of chronic health conditions such as hypertension and diabetes [2].

The objective of exercise prescription is to assess current levels of activity and guide patients as they increase their levels of exercise. A total of 150 min of MVPA per week is required to sustain health, whereas 300 min is needed to improve health [3]. It is rare for any information on physical activity to be captured in clinical practice, and when patients are assessed for their level of activity, little emphasis is placed on objective measures. In the domain of population research, physical activity questionnaires have for many years been the primary means of measurement [4,5], although more recent studies have used wearable devices to assess activity [6,7].

Today, objective measurement of individual physical activity under free-living conditions has become a reality following the introduction of miniaturized step counters and triaxial accelerometer technology. What started off as ball-in-a-box devices that were clipped to the belt have evolved into sleek wrist-worn gadgets that connect wirelessly to mobile phones and the Internet. Data are captured, logged, analyzed, and displayed within a matter of seconds. Wearable devices, which once had only been used in research settings, are now being marketed directly to consumers on a large scale. A recent systematic review of 22 studies has demonstrated high validity of step count measures among commercial devices [8]. Our own experience has shown remarkable correlation between wrist-worn step counters and scientific devices under free-living conditions [9]. It therefore follows that health care providers and exercise professionals might learn to review and interpret the large amounts of objective physical activity data that patients and clients may have collected incidentally.

Apart from recording daily step counts or total volumes of physical activity, a number of commercial wrist-worn trackers and hybrid watches feature optical sensors that estimate heart rates by means of photoplethysmography. The noninvasive optical probe detects the small variation in light absorption brought about by pulsatile perfusion of tissues [10]. In principle, heart rate measures should offer a number of advantages in activity tracking. First, heart rate monitors outperform accelerometers in capturing non–weight-bearing activities such as cycling and rowing. Second, whereas the latter reflects total volume of activity, heart rate monitors provide information on the relative intensity of activity, allowing MVPA to be more accurately discerned from light activity. Finally, where information on real-time relative intensity is available, there are, in theory, potential applications in the realm of safety monitoring for users at risk of overtraining [11]. The current approach to intensity assessment still relies on tactile carotid or radial pulse rates, a method which is potentially cumbersome or inaccurate in laypersons.

Naturally, there are some important disadvantages using heart rates to approximate physical activity. Heart rate is a vital sign which responds to a multitude of physiological stimuli, including emotional state and illness. Heart rates also tend to exhibit considerable lag in the minutes following the cessation of activity. Moreover, devices that have been shown to track heart rates reliably, such as Polar [12,13] and Actiheart [14,15], by measuring myocardial electric potentials (akin to an electrocardiogram), are cumbersome to wear over extended periods as they need to be strapped across the chest.

We sought to explore whether it would be feasible to adopt a wrist-worn consumer wearable to augment health promotion strategies such as exercise prescription. By monitoring heart rate information through the wearable device, the patient would have a more convenient means to guide the calibration of intensity to attain a specific training target [11]. In turn, these measures could be useful to review compliance during follow-up appointments with the health care provider and make available objective feedback to inform behavior change strategies [16].

The literature offers conflicting information on the utility of wrist-worn heart rate sensors when tested for validity while participating in exercise protocols under laboratory conditions. Authors who chose to cite strong correlation coefficients and low mean percentage errors as validation criteria concluded that wrist-worn devices performed well [17-19]. Others with a stricter definition of accuracy choosing to examine mean bias and levels of agreement [20] concluded that devices performed inadequately. When validated in hospital patients, the devices were found suitable for a subset of patients who were in sinus rhythm [21]. We found only a limited number of validation studies that collected information on minute-by-minute heart rates [22] and daily energy expenditure [23] outside the laboratory. In the time following our data collection efforts, a class action lawsuit was filed against Fitbit Inc, alleging that the devices “consistently mis-record heart rates by a very significant margin, particularly during exercise” [24].

To address some of the existing gaps in the literature, we have conducted this validation study to assess the accuracy of a common wrist-worn heart rate tracking device under free-living conditions and to evaluate the feasibility of including heart rate tracking measures as part of population-based activity monitoring and mobile health interventions.

## Methods

### Participants

We aimed to recruit 40 members of the university’s staff and students through department-approved internal emails for a pilot study assessing the feasibility of wearable-based observational studies examining physical activity, nutrition, and mental well-being. Out of this pilot study group, a convenience sample of 10 participants would be invited to participate in an additional validation component to examine the accuracy of heart rate measures provided by Fitbit Charge HR (Fitbit, San Francisco CA, USA). Participants could be included if they owned a compatible mobile phone with a data plan, were aged between 18 and 65 years, and were unlikely to travel abroad over the subsequent 1 month. The following criteria excluded an interested participant from the study: having a severe medical condition that would prevent participation in physical activity, discomfort, or unwillingness to wear multiple devices concurrently and participation in activities or work that would restrict the use of the devices.

### Study Procedures and Data Collection

We compiled baseline characteristics for all our participants by means of a self-administered questionnaire. Measures of height and weight were taken using a SECA stadiometer (SECA GmbH, Hamburg, Germany) at our study site.

Each participant was provided a new Fitbit Charge HR (Fitbit) tracker to be worn on the nondominant hand throughout the pilot study. Participants were instructed in the use of the tracker device and the installation of mobile phone apps according to manufacturer’s specifications. They were also taught to synchronize the Fitbit tracker periodically.

Each of the 10 participants in the heart rate validation series were also fitted with one Polar H6 heart rate monitor (Polar Electro Oy, Kempele, Finland) worn across the chest. To record the Polar H6 heart rate monitor (Polar) data, these participants were provided with an Actigraph GT3X+ logger (Actigraph) on Bluetooth receiver mode set to sample measures at 10-s intervals and worn on the same wrist as the Fitbit device. The 10 participants were asked to wear all 3 devices for at least 3 and at most 6 continuous hours of nonsleeping activities. They were encouraged to continue pursuing their usual activities, excluding water sports. The Polar and wrist-worn Actigraph devices could be removed before bedtime and returned to the study site over the following days. Participants would continue to use their Fitbit trackers for the remaining 1 month of free-living study. Participants used their personal data plans to run synchronizations with the Fitbit server.

Fitbit heart rate measures were downloaded directly from the Web server using a developer’s application programming interface (API) issued by Fitbit. Polar measures were collated from the wrist-worn Actigraph devices. Common wear time for the validation study was defined as every 1-min epoch, which reflected a nonzero heart rate on both devices. For the 1-month period of continuous monitoring, any nonzero heart rate registered by the Fitbit was defined as valid 1-min epoch of wear time. A valid day of wear time was defined as having at least 600 1-min epochs of nonzero counts within 1 calendar day.

### Graphical Analysis

Our first dataset comprised one Fitbit heart rate measure for each discreet 1-min epoch, whereas six 10-s measures for the same epoch were available from the Polar device. While the Fitbit API allowed us to review heart rate measures at intervals less than 1 min, the time differences between measures were irregular. Given that Fitbit users would only have access to data logs recorded by minute, we did not attempt to generate our own summary measures for within-minute heart rates. It is worth noting that the literature recommend that photoplethysmographic readings should be averaged over a 60-s duration to obtain a reliable measure [10]. To appreciate the data contributed by individual participants, we generated time series plots of discreet 1-min epochs where Fitbit measures were superimposed onto ranges of Polar 10-s measures. Thereafter, 10-s Polar heart rate measures were averaged for each 1-min epoch. Subsequently, we rank-ordered aggregated discreet epochs by their average Polar measure, divided these epochs by deciles, and constructed box plots to compare average Polar and Fitbit measures. Box plots for the width of within-epoch ranges of 10-s Polar measures were included in this descriptive plot.

### Statistical Analysis

Intraclass correlation coefficients (ICCs) were calculated using a mixed effects model assessing for absolute agreement between average 10-s Polar measures, and 1-min Fitbit measures. ICCs were calculated first for overall measures and then for measures stratified by physical activity heart rate zone cut-offs proposed by the Centers for Disease Control and Prevention [25] and by individual participants. Given that the Polar device was chosen as reference, heart rate zones were assigned according to each participant’s age and average 10-s Polar measure within discreet epochs. Two Bland-Altman plots were constructed to visually evaluate the overall differences in absolute measures within heart rate zones. A two-by-two table was constructed to estimate the sensitivity, specificity, and positive and negative predictive values for Fitbit devices, correctly identifying MVPA heart rate zones where average 10-s Polar values were considered as reference.

In our second dataset, we compiled all Fitbit heart rate measures obtained in the 1-month free-living study period. The aggregated valid days and epochs of wear time were compiled in a bar chart with superimposed dot and whiskers plots. We tabulated summary statistics for each participant, detailing the number of 1-min epochs spent in respective heart rate zones under free-living conditions.

All statistical analyses were conducted using STATA (Version 13.1, StataCorp LP). Given the exploratory nature of this study, *P*<.05 was chosen as a level of statistical significance. The strength of ICC coefficients was interpreted based on the following definitions: weak (*r*<.5), moderate (.5-.7), and strong (*r*>.7). This study was approved by the institutional review board of the National University of Singapore.

## Results

### Study Participants

From our pilot study group of 20 males and 20 females, we recruited 3 females and 7 males to participate in the heart rate validation segment. Recruitment and data collection began on November 4, 2015, and the last day of assessment was January 7, 2016. Nine out of 10 participants were students, and their average body mass index was 22.9 kg/m^2^ (standard deviation [SD] 3.8). Table 1 describes the characteristics of the final sample of 10 study participants.

**Table 1:** Sample characteristics (N=10).

Characteristics		Value (N=10)
**Gender, n**		
	Female	3
	Male	7
**Occupation, n**		
	Undergraduate	3
	Postgraduate	6
	Staff	1
Age in years, mean (SD^a^)		25.4 (3.7)
Body mass index in kg/m^2^, mean (SD)		22.9 (3.8)
**Number of valid 1-min epochs contributed, mean (SD)**		
	Paired measures	250 (95)
	1-month follow-up period	38880 (11758)

^a^SD: standard deviation.

**Figure 1:**
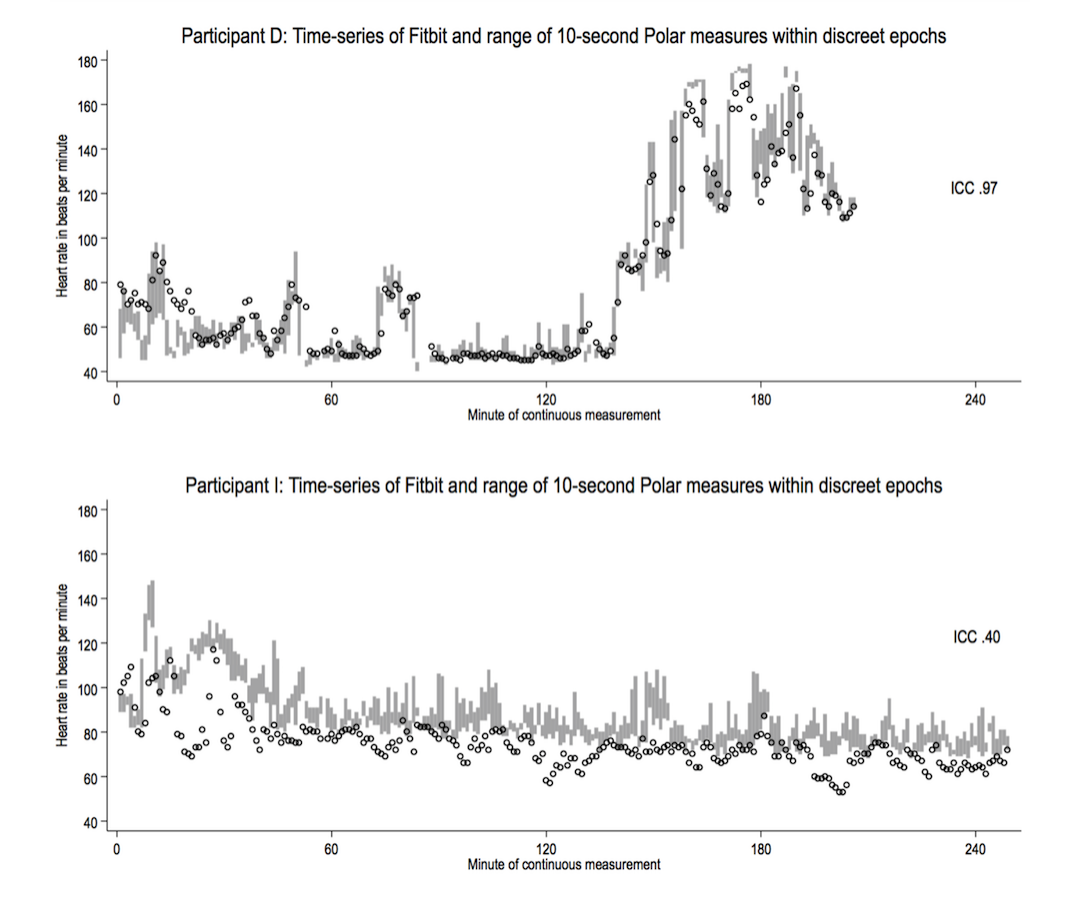
Time-series of Fitbit 1-min measures (circles) and Polar 1-min ranges (gray bars) estimating heart rate in beats per minute for participants D and I.

### Validation of Fitbit Measures Compared With Polar

Of a total of 2769 possible 1-min epochs, 2509 valid paired readings were identified, with each participant contributing on average 250 (SD 95) epochs. Unpaired readings were treated as missing data and omitted from further analyses. For illustrative purpose, 2 of the 10 time series plots are shown in Figure 1, representing the strongest and weakest measures of intraclass correlation.

**Figure 2:**
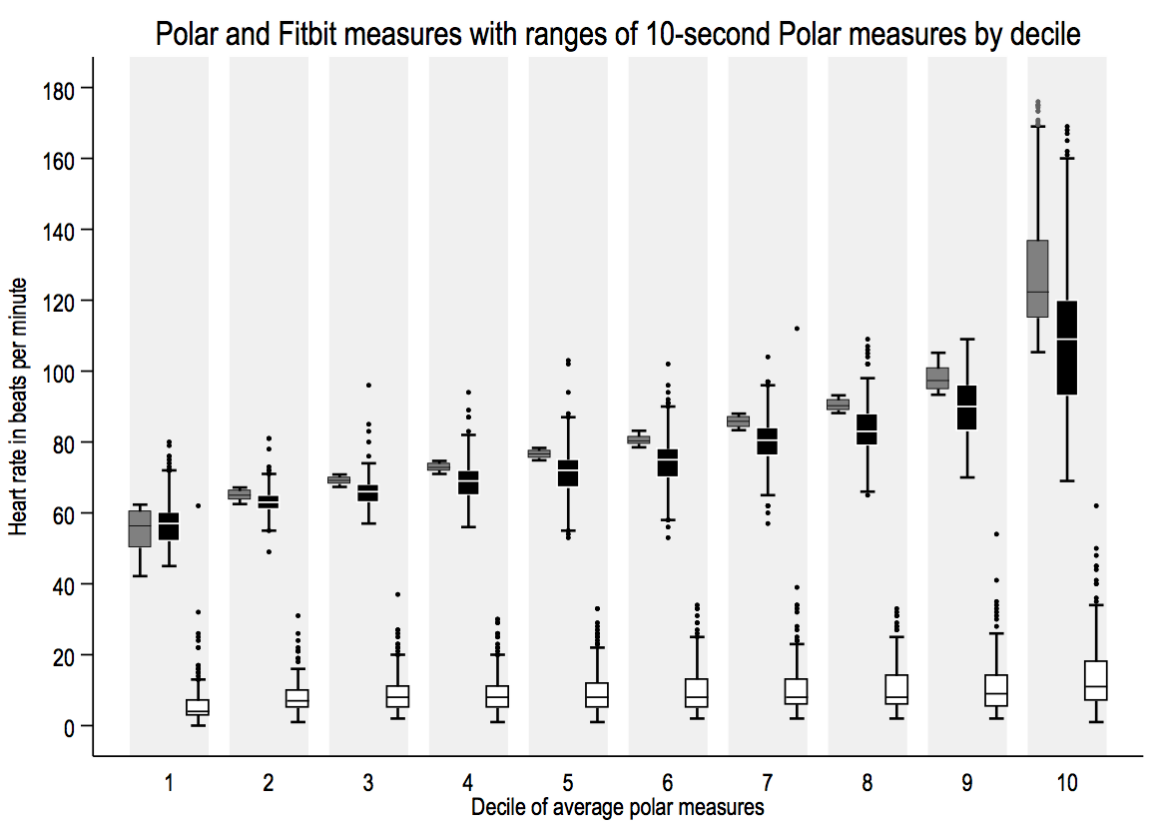
Box-plots providing by-decile comparisons of average Polar (dark gray) and Fitbit (black) measures, width of within-epoch ranges of 10-second Polar measures (white) included (n=2509).

The graphical comparison of aggregated epochs in Figure 2 shows how heart rate measures from Fitbit were consistently lower than Polar, whereas the width of 10-s Polar value ranges remained consistent.

**Figure 3:**
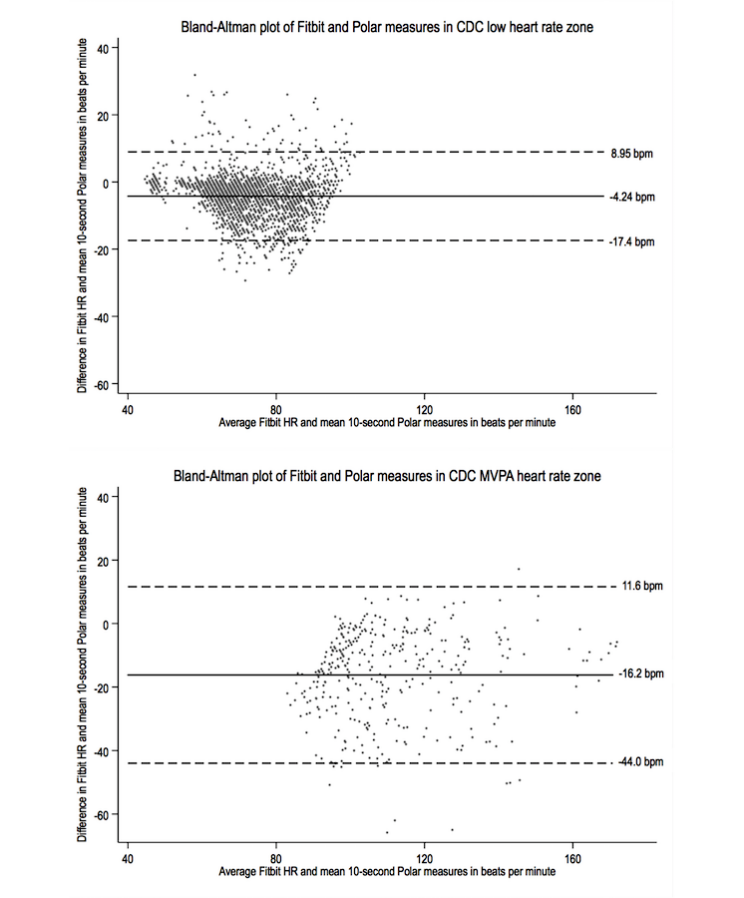
Bland-Altman plots for paired measures in low (top) and moderate to vigorous physical activity (MVPA; bottom) Centers for Disease Control and Prevention (CDC) heart rate zones.

Table 2 shows the ICCs and differences between Fitbit and Polar measures. The overall ICC between both devices was strong (.83; 95% CI 0.63-0.91) and ranged from .40 to .97 across participants. The ICC was markedly weaker at MVPA heart rate zones as compared with the low heart rate zone. On average, Fitbit devices measured heart rates that were −5.96 bpm (95% CI −6.33 to −5.60) lower than Polar. Reviewing the differences between participants, we noted that the underestimate was statistically significant in all but 1 participant, who also demonstrated the strongest ICC. Again, the difference between both devices was greater in MVPA heart rate zones. This finding was reproduced in the Bland-Altman plots of measures (Figure 3).

**Table 2:** Overall and stratified comparisons between Fitbit and Polar of minute-by-minute epochs.

1-min epochs		n	Within-epoch comparison of 1-min Fitbit and average 10-s Polar heart rates (in bpm)		
			Intraclass correlation coefficient^a^ (95% CI)	Average difference^b^ (95% CI)	*P* value^b^
Overall		2509	.83 (0.63-0.91)	−5.96 (−6.33 to −5.60)	<.001
**By Centers for Disease Control and Prevention heart rate zone^c^**					
	Low (<50%)	2146	.77 (0.55-0.87)	−4.24 (−4.53 to −3.96)	<.001
	Moderate to vigorous physical activity (≥50%)	363	.56 (0-0.79)	−16.2 (−17.6 to −14.7)	<.001
**By participant**					
	A	348	.87 (0.69-0.94)	−2.58 (−2.99 to −2.18)	<.001
	B	358	.75 (0.50-0.86)	−3.92 (−4.55 to −3.29)	<.001
	C	169	.70 (0.28-0.85)	−5.08 (−6.01 to −4.14)	<.001
	D	198	.97 (0.96-0.98)	0.25 (−1.08 to 1.58)	.70
	E	359	.62 (0.14-0.81)	−6.40 (−7.13 to −5.67)	<.001
	F	208	.47 (0-0.74)	−17.4 (−19.5 to −15.4)	<.001
	G	320	.72 (0.19-0.88)	−5.05 (−5.62 to −4.47)	<.001
	H	228	.81 (0.65-0.88)	−5.20 (−6.45 to −3.94)	<.001
	I	248	.40 (0-0.68)	−12.3 (−13.6 to −11.1)	<.001
	J	73	.93 (0.88-0.95)	−0.92 (−1.69 to −0.14)	.02
					

^a^ICCs derived using two-way mixed effects model for absolute agreement.

^b^Statistics derived in paired *t* tests.

^c^Calculated as percent of maximal heart rate (220 bpm—age in years) for discreet 1-min epochs drawing from the average 10-s Polar measure and age of the respective participant.

Within the aggregate 2509 1-min epochs shown in Table 3, only 363 were spent in the MVPA heart rate zone according to Polar measures.

### One-Month Continuous Observation

**Figure 4:**
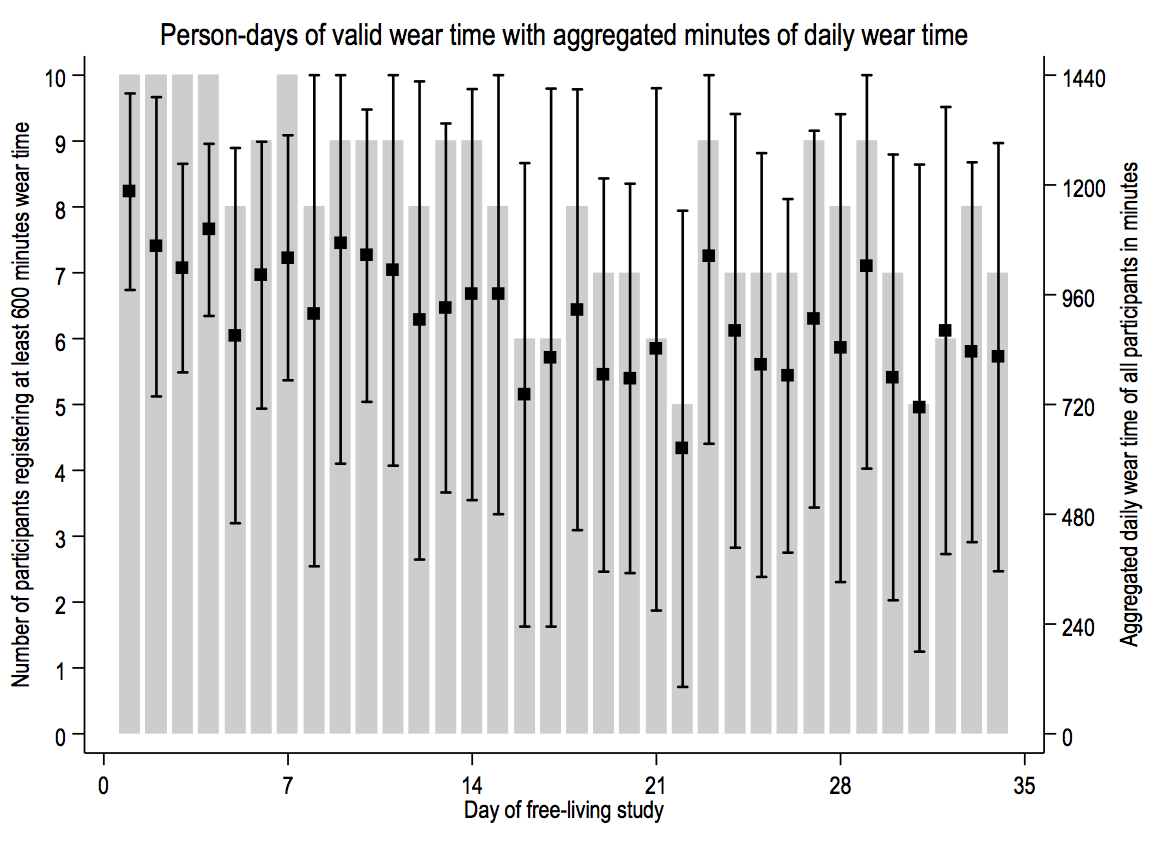
Number of participants with valid days (gray bars) with distribution of aggregated wear time means (boxes) and one standard deviation (whiskers).

Figure 4 shows that wear time on valid days was consistent, although the number of participants providing valid days of device usage declined over the course of the study period.

On valid days of device usage as shown in Table 4, 24.4% of epochs were classified as nonwear time, 71.7% of epochs fell within the low intensity heart rate zone, and 3.9% of epochs were classified as MVPA. Thus, on average, participants spent 55 min (SD 34) per day in the MVPA heart rate zone.

**Table 3:** Number of 1-min epochs spent in Centers for Disease Control and Prevention (CDC) physical activity heart rate zone; n=2509 paired observations. Sensitivity and specificity were 52.9% (192/363) and 98.6% (2115/2146), respectively. The positive predictive value for a 1-min epoch categorized as MVPA by Fitbit values was 86.1% (192/223), and the negative predictive value was 92.52% (2115/2286).

Epoch categorization		According to Fitbit values, n (%)		Total, n (%)
		Low	MVPA^a^	
**According to average 10-s Polar values**				
	Low	2115 (98.6)	31 (1.4)	2146 (100.0)
	MVPA	171 (47.1)	192 (52.9)	363 (100.0)

^a^MVPA: moderate to vigorous physical activity.

**Table 4:** Free-living data collection. Over n=270 valid person-days of Fitbit usage.

Device use		Valid days	Total number of epochs	Number of 1-min epochs spent in CDC^a^ physical activity heart rate zone, n (%)			Average daily minutes spent in MVPA^b^ heart rate zone
				No valid reading	Low	MVPA	
Overall		270	388,800	94,993 (24.4)	278,856 (71.7)	14,951 (3.9)	55 (SD 34)
**By participant**							
	A	21	30,240	13212 (43.7)	16,397 (54.2)	631 (2.09)	30
	B	30	43,200	12076 (28.0)	29,729 (68.8)	1395 (3.2)	47
	C	26	37,440	13984 (37.4)	20,691 (55.3)	2765 (7.4)	106
	D	33	47,520	16136 (34.0)	30,600(64.4)	784 (1.7)	24
	E	22	31,680	6754 (21.3)	23,686 (74.8)	1240 (3.9)	56
	F	32	46,080	10703 (23.2)	33,593 (72.9)	1784 (3.9)	56
	G	34	48,960	2743 (5.6)	44,580 (91.1)	1637 (3.3)	48
	H	34	48,960	2694 (5.5)	45,488 (92.9)	778 (1.6)	23
	I	30	43,200	11901 (27.6)	27,614 (63.9)	3685 (8.5)	123
	J	8	11,520	4790 (41.6)	6478 (56.2)	252 (2.2)	32

^a^CDC: Centers for Disease Control and Prevention.

^b^MVPA: moderate to vigorous physical activity.

## Discussion

### Principal Findings

In this free-living validation study, we have compiled rich data by relatively convenient means, capturing 2509 1-min epochs of paired data and tens of thousands of unpaired 1-min epochs in the follow-up period. Visual inspection of within-participant heart rate plots showed that there were differences in how well Fitbit readings coincided with Polar ranges. We ascertained an overall strong ICC for absolute agreement between Fitbit and Polar measures that varied markedly between participants and diminished at heart rates that represent moderate to vigorous intensity physical activity. The Fitbit devices identified just over half of MVPA heart rate zone readings correctly.

Our summary measure of a 6 bpm or 7% underestimate of heart rates measured by Fitbit was in keeping with current literature where error estimates have been established under laboratory conditions [18,26,27]. In MVPA heart rate zones, we found an average underestimate of 16 bpm, which was shown to markedly impact Fitbit’s ability to correctly identify time spent in MVPA under free-living conditions. This finding of a larger error at higher heart rates is consistent with other studies [26,28]. However, our findings suggest that the systematic underestimation of heart rates might partially be accounted for by differences between participants.

Applying our own measures of sensitivity and specificity to the data obtained during the 1-month follow-up period, we could surmise that close to an hour’s worth of MVPA epochs were not captured on any given day. This underestimate of daily MVPA time contrasts with the overestimate observed in a free-living validation study of a wrist-worn tracker of the same brand that measured bouts of activity based on accelerometry [29]. It is also important to note that Fitbit wear time and the number of participants wearing it sufficiently long had decreased considerably even over the 1-month monitoring period. This is consistent with findings from other studies [30] that have also reported considerable drops in compliance with wearable device use over time.

Our free-living validation study into the accuracy of wrist-worn heart rate monitors has several implications on their potential usefulness in monitoring relevant physiological parameters over time and tracking compliance with exercise prescriptions. The results are in keeping with the past studies, which concluded that the device would fare poorly in the calibration of intensity of activity owing to insufficient accuracy. Concerning activity tracking, we found that the devices would fail to recognize one in two MVPA heart rate zone epochs, thus diminishing their value as a means of assessing activity levels objectively. Our follow-up data suggest that device use declined over the course of the study, further complicating potential uses as a compliance monitoring tool.

Overall, our findings have demonstrated that more emphasis should be placed on eliminating systematic error in the tracker measures. Our data showed that errors might be explained in part by putative between-participant differences that would include device fit and skin surface characteristics. Additional mathematical calibration might be appropriate for the trackers to more reliably detect MVPA heart rate zones. As these sensors become increasingly ubiquitous, their potential role in exercise prescription and health promotion merits further evaluation.

### Limitations

Our study design was limited to a small sample of mostly male, young adults who did not report significant health issues, thus limiting the generalizability of our findings. The recruitment of female participants was affected by expressed discomfort wearing the Polar H6 chest strap beneath undergarments. In addition, the measurement period was restricted over a few hours of one day where participants would engage in their normal activities. Although this provided us the necessary information on usual day-to-day life, it resulted in a limited number of paired measures, particularly in the moderate to vigorous heart rate zones. We did not include any form of activity diary in the 1-month period of follow-up, thus limiting our ability to verify the total duration of MVPA accrued. The sensitivity of the photoplethysmographic probe is strongly affected by placement and skin condition [10]. Although we provided advice on proper fit, it is plausible that participants may have foregone scientific accuracy in favor of personal comfort by loosening wrist straps. Due to the logistics of the study, we were unable to ascertain proper fit at the end of the observation period, which could have provided further insights into the apparent interpersonal differences in Fitbit accuracy. Finally, it is important to note that the optical sensors provide a measure of microvascular perfusion, whereas our reference device registers myocardial electric potentials. In practice the arterial pulse rate is often synonymous with the rate of cardiac contractions, but this level of equivalence might be considered inappropriate in the evaluation of photoplethysmographic devices, which are known to be affected by movement and other artifacts that contribute to error rates of up to 8% [10].

### Conclusions

The nature of this study was part validation and part exploration. While the overall ICC for absolute agreement appears strong, our data suggest that under free-living conditions, Fitbit Charge HR trackers overall compared poorly against the reference device, especially at higher heart rate zones. Our findings are in line with findings of past studies, which have expressed concern that such devices might not provide adequate information to guide exercise intensity or detect MVPA. Given the nature of our small pilot study with a limited period of observation, further research with a larger sample is warranted to confirm our results.

## Abbreviations

API: application programming interface
CDC: Centers for Disease Control and Prevention
ICC: intraclass correlation coefficient
MVPA: moderate to vigorous physical activity
SD: standard deviation
SE: standard error
